# Editorial for the Special Issue on Micro-Electro Discharge Machining: Principles, Recent Advancements and Applications

**DOI:** 10.3390/mi12050554

**Published:** 2021-05-13

**Authors:** Irene Fassi, Francesco Modica

**Affiliations:** 1 STIIMA CNR, Institute of Intelligent Industrial Technologies and Systems for Advanced Manufacturing, National Research Council, Via A. Corti 12, 20133 Milan, Italy; 2 STIIMA CNR, Institute of Intelligent Industrial Technologies and Systems for Advanced Manufacturing, National Research Council, Via P. Lembo 38/F, 70124 Bari, Italy

Micro Electrical Discharge Machining (micro-EDM) is a thermo-electric and contactless process most suited for micro-manufacturing and high-precision machining, especially when difficult-to-cut materials, such as super alloys, composites, and electro conductive ceramics, are processed. Many industrial domains exploit this technology to fabricate highly demanding components, such as high-aspect-ratio micro holes for fuel injectors, high-precision molds, and biomedical parts.

Moreover, the continuous trend towards miniaturization and high precision functional components boosted the development of control strategies and optimization methodologies specifically suited to address the challenges in micro- and nano-scale fabrication.

This Special Issue showcases 12 research papers and a review article focusing on novel methodological developments on several aspects of Micro Electrical Discharge Machining: machinability studies of hard materials (TiNi Shape Memory Alloys, Si_3_N_4_–TiN ceramic composite, ZrB_2_-Based Ceramics Reinforced with SiC Fibers and Whiskers, Tungsten-cemented carbide, Ti-6Al-4V alloy, Duplex Stainless Steel and Cubic boron nitride), process optimization adopting different dielectrics or electrodes, characterization of mechanical performance of processed surface, process analysis and optimization via discharge pulse-type discrimination, hybrid processes, fabrication of molds for inflatable soft microactuators, and implementation of low-cost desktop micro-EDM system.

In further detail, Zhao et al. [1] have investigated Laser-Assisted Electrochemical Discharge Machining for micro-processing of insulating and hard-brittle materials typified by glass. This study compared morphological features obtained by means of single processing and hybrid processing methods concerning the fabrication of microgrooves on quartz workpiece. The combination of these two methods allows to transform gradually V-shaped microgrooves into U-shaped microgrooves. Micro Electrical Discharge Machining has been characterized by Zhu et al. [2] for Surface Modification of TiNi Shape Memory Alloys using a TiC Powder Dielectric. The study reveals that TiC powder addition with a concentration of 5 g/L had a positive effect on increasing the electro-discharge frequency and MRR, reducing the surface roughness. The machined surface presents a recast layer with good adhesion and high hardness, due to metallurgical bonding, and contains CuO_2_, TiO_2_, and TiC phases, contributing to an increase in the surface microhardness from 258.5 to 438.7 HV, which could be beneficial for wear resistance in biomedical orthodontic applications. In accordance with potential effects on mechanical properties and oxidation performance, a study on ZrB_2_-Based Ceramics reinforced with SiC Fibers and whiskers has been performed by Quarto et al. [3] By means of the pulse-type characterization, results indicated how reinforcement shapes affect the energy efficiency of the process and change the surface aspect. The microEDM milling machinability of the Si_3_N_4_–TiN ceramic composite was investigated through the discharge pulse-type discrimination algorithm evaluating the material removal rate, tool wear ratio and surface quality in [4] by Marrocco et al. The analysis shows that MRR was sensitive only to normal pulses, while the occurrence of arcs and delayed pulses induced unexpected improvements in tool wear. Additionally, the inspection of the surface, performed by SEM and EDS analyses, showed the presence of re-solidified droplets and micro-cracks, which modified the chemical composition and the consequent surface quality of the machined micro-features. Ablyaz et al. [5] have developed a Surface Characterization and a Tribological Performance Analysis of Electric Discharge Machined Duplex Stainless Steel considering the main process parameters and three variants of electrode material (Graphite, Copper–Tungsten and Tungsten). The results revealed that the machined surface at high spark energy in EDM oil portrayed porosity, oxide formation, and intermetallic compounds, and then exhibited 70% superior wear resistance compared to the un-machined sample and contextually improving surface wettability.

The machinability via Electrodischarge Drilling of microholes with 410 μm in diameter and 1 mm deep in difficult-to-cut cubic boron nitride (c-BN) material was studied by Wyszynski et al. [6]. Tests were executed using tungsten electrode tool in Exxol80 (hydrocarbon oil) and varying pulse on and off times, pulse frequency and working current. Results show that relatively high working current and short pulses improve machining quality. Wire electrical discharge grinding (WEDG) is adopted by Milana et al. [7] for shaping several molds to cast rubber and create complex void for inflatable soft microactuators. All microactuators that have a cylindrical shape with a length of 8 mm and a diameter of 0.8 mm have been characterized and tested showing that complex deformation patterns, such as curling, differential bending or multi-points bending, can be achieved.

In order to deal with the high wear ratio bottleneck of micro-electrode tool, Huang et al. [8] have investigated the use of a liquid droplet suspended at the end of a capillary nozzle as an electrode. The liquid supply, the geometry and shape control of the tip were analyzed both via simulation and experimentation showing the feasibility of the method. The feasibility of a novel filling method to fabricate a composite 3D microelectrode for processing 3D microstructures via micro-EDM without steps is analyzed theoretically and experimentally by Lei et al. [9]. Wear and corrosion resistance of the Ti-6Al-4V alloy processed by EDM using multi-walled carbon nanotubes (MWCNTs) mixed with dielectric were experimentally investigated by Singh et al. [10]. The study reveals that the formation of intermetallic compounds, oxides, and carbides allows a dramatic enhancement of the wear-resistant and the corrosion protection efficiency up to 95%, and 96.63%, respectively, in comparison to using plan dielectric. Xu et al. [11] have proposed foil queue microelectrodes with tapered structures in order to solve the typical step effect issue when these microelectrodes are used for eroding 3D microstructure. The influence of the taper angle and the number of microelectrodes on the step effect were investigated and tested, reporting that the step effect on the 3D microstructure’s surface became less evident when the taper angle and the number of foils–microelectrodes increase.

Wu et al. [12] have presented a low-cost desktop micro-EDM system for fabricating micro-holes in tungsten-cemented carbide materials reporting experimental test of micro-holes with a diameter of 0.07 mm and thickness of 1.0 mm drilled with a cut-side shaped micro-tool.

Finally, with the aim of helping researchers for selecting suitable powders and identifying different aspects of powder-mixed dielectric fluid of EDM, a paper review is presented by Abdudeen et al. [13]. The paper discusses and compares studies about various powders used for the process focusing on achieving a more efficient metal removal rate, reduction in tool wear, and improved surface quality of the powder-mixed EDM process.

We thank all the authors who submitted their papers to this Special Issue “Micro-Electro Discharge Machining: Principles, Recent Advancements and Applications”. We would also like to acknowledge the reviewers who carefully reviewed all the submitted papers dedicating their time and helping to improve the quality of this Special Issue. 
